# Biopsy for advanced hepatocellular carcinoma: results of a multicentre UK audit

**DOI:** 10.1038/s41416-021-01535-2

**Published:** 2021-09-15

**Authors:** Alexa Childs, Nekisa Zakeri, Yuk Ting Ma, Joanne O’Rourke, Paul Ross, Essam Hashem, Richard A. Hubner, Kimberley Hockenhull, Chinenye Iwuji, Sam Khan, Daniel H. Palmer, Joanna Connor, Daniel Swinson, Suzanne Darby, Chiara Braconi, Tom Roques, Dominic Yu, Tu Vinh Luong, Tim Meyer

**Affiliations:** 1 Department of Oncology, Royal Free London NHS Foundation Trust, London, UK; 2 University Hospitals Birmingham NHS Foundation Trust, Birmingham, UK; 3 King’s College Hospital NHS Foundation Trust, London, UK; 4 Department of Medical Oncology, The Christie NHS Foundation Trust, Manchester, UK; 5 Oncology Department, University Hospitals of Leicester NHS Trust, Leicester, UK; 6 University of Liverpool and The Clatterbridge Cancer Centre NHS Foundation Trust, Liverpool, UK; 7 Leeds Cancer Centre, St James’s University Hospital, Leeds Teaching Hospitals NHS Trust, Leeds, UK; 8 Sheffield Teaching Hospitals NHS Foundation Trust, Sheffield, UK; 9 The Royal Marsden NHS Foundation Trust, London, UK; 10 Norfolk and Norwich University Hospitals NHS Foundation Trust, Norwich, UK; 11 Department of Radiology, Royal Free London NHS Foundation Trust, London, UK; 12 Department of Cellular Pathology, Royal Free London NHS Foundation Trust, London, UK; 13 UCL Cancer Institute, University College London, London, UK

**Keywords:** Hepatocellular carcinoma, Hepatocellular carcinoma

## Abstract

**Background:**

Advanced hepatocellular carcinoma (HCC) is commonly diagnosed using non-invasive radiological criteria (NIRC) defined by the European Association for the Study of the Liver or the American Association for the Study of Liver Diseases. In 2017, The National Institute for Clinical Excellence mandated histological confirmation of disease to authorise the use of sorafenib in the UK.

**Methods:**

This was a prospective multicentre audit in which patients suitable for sorafenib were identified at multidisciplinary meetings. The primary analysis cohort (PAC) was defined by the presence of Child-Pugh class A liver disease and performance status 0–2. Clinical, radiological and histological data were reported locally and collected on a standardised case report form.

**Results:**

Eleven centres reported 418 cases, of which 361 comprised the PAC. Overall, 76% had chronic liver disease and 66% were cirrhotic. The diagnostic imaging was computed tomography in 71%, magnetic resonance imaging in 27% and 2% had both. Pre-existing histology was available in 45 patients and 270 underwent a new biopsy, which confirmed HCC in 93.4%. Alternative histological diagnoses included cholangiocarcinoma (CC) and combined HCC-CC. In cirrhotic patients, NIRC criteria had a sensitivity of 65.4% and a positive predictive value of 91.4% to detect HCC. Two patients (0.7%) experienced mild post-biopsy bleeding.

**Conclusion:**

The diagnostic biopsy is safe and feasible for most patients eligible for systemic therapy

## Background

The non-invasive radiological criteria (NIRC) for the diagnosis of hepatocellular carcinoma (HCC) were first defined by the European Association for the Study of the Liver (EASL) in 2001, but have been refined over time [1]. In the current version of the guidelines, a diagnosis of HCC can be made when a nodule ≥1 cm occurring in a cirrhotic liver displays both arterial phase hyperenhancement and washout on the portal venous or delayed phase using multiphasic computed tomography (CT) or dynamic contrast-enhanced magnetic resonance imaging (MRI) [2]. The American Association for the Study of Liver Diseases (AASLD) also define the diagnosis of HCC by these criteria, but additionally consider threshold growth as described by Liver Imaging Reporting and Data System (LI-RADS) [3]. The majority of studies investigating the diagnostic accuracy of NIRC are retrospective and the focus has been on the detection of early disease [4, 5]. Despite this, the diagnostic criteria have been extended to advanced disease with the consequence that patients with HCC usually embark on systemic therapy without a histological diagnosis. In this respect, HCC is unique among solid tumours and, in the era of precision medicine, HCC research has been disadvantaged by the lack of archival tissue from patients with advanced disease. Arguments advanced against biopsy include the risk of tumour seeding and bleeding. However, there remain very limited data on the diagnostic accuracy of imaging and the complication rate associated with biopsy in patients with advanced disease.

In November 2017, NHS England mandated histological confirmation of HCC prior to the initiation of sorafenib. This was based on the fact that pathological diagnosis was required for the SHARP trial that led to the registration of sorafenib [6]. The requirement for biopsy was only waived in exceptional circumstances where a biopsy was deemed to be too high risk or technically not feasible in the opinion of the specialist HCC multidisciplinary team (MDT) meeting in a patient who otherwise met the non-invasive diagnostic criteria of HCC. The requirement for biopsy permitted the conduct of a multicentre, prospective audit to establish the safety and outcome of biopsy in patients with suspected advanced HCC.

## Methods

Eleven UK centres providing specialist care for patients with liver cancer agreed to participate and were provided with a standardised report form to enable the prospective collection of anonymised data. Patients were included if they were deemed suitable for systemic therapy for advanced HCC at a dedicated MDT meeting and subsequently assessed in the local clinic. Patient characteristics including age, sex, disease aetiology, presence of cirrhosis, alpha-fetoprotein (AFP) and Child-Pugh score were recorded. Contrast-enhanced CT and/or MRI was performed, and centres were required to report the presence of arterial enhancement, washout in the portal venous phase and radiological evidence of chronic liver disease as evidenced by liver contour, varices and splenomegaly. In addition, the number of tumours, size of largest tumour, presence of macrovascular invasion and extrahepatic spread were also documented. In the absence of previous histology or perceived high risk of biopsy, percutaneous tumour biopsy was performed and histology was reported locally. The primary analysis cohort (PAC) comprised all those who met the criteria for sorafenib as defined by National Institute for Clinical Excellence (NICE) guidelines, namely Child-Pugh class A liver disease and performance status (PS) ≤2. However, all patients were included in the safety analysis. This study was classified as a national audit and clinical evaluation by the Caldecott Guardian, the primary aim of which was to evaluate the feasibility, safety and outcome of diagnostic liver biopsy. As such, individual patient consent was not required. The study was reported according to Standards for Reporting Diagnostic accuracy studies (STARD) guidelines.

## Results

### Patient characteristics

**Fig. 1:**
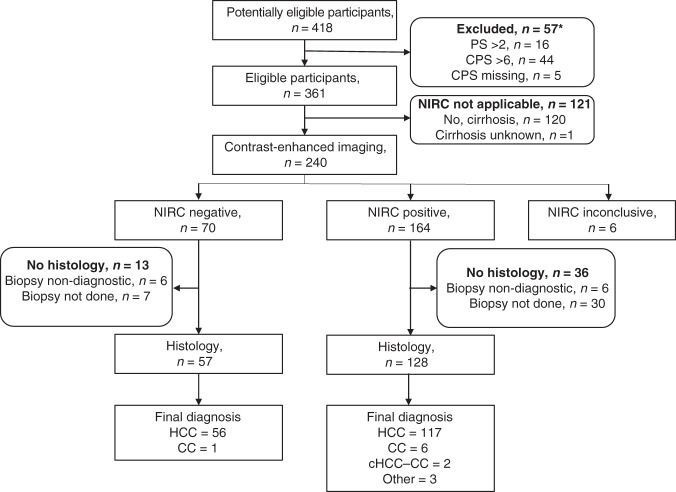
STARD flow diagram. *Eight patients had more than one reason. PS performance status, CPS Child-Pugh score.

Overall, 11 UK hospitals contributed to this audit and data were submitted on 418 patients identified between January 2018 and August 2020. Fifty-seven patients who were not confirmed to be Child-Pugh class A and PS ≤2 were excluded, leaving a total of 361 for the PAC (see STARD flow diagram in Fig. 1 and Table 1). The median age was 68, the majority were male and 83% had a PS of 0 or 1. Chronic liver disease was present in 76% with HCV, alcohol and NASH/NAFLD accounting for the majority, each being present in broadly equivalent proportion. Sixty-six per cent of patients were cirrhotic. More than one risk factor for chronic liver disease was present in 12%. At least one previous locoregional or surgical intervention had been delivered in 43%, with the most common being arterial embolic treatment, and 11% had received at least two transarterial embolisation/transarterial chemoembolisation procedures. AFP was ≥400 ng/mL in 37%.

**Table 1:** Patient characteristics.

Characteristic	*n* = 361	%
Median age (range)	68 (21–87)	
Gender		
Male	296	82
Female	65	18
Performance status		
0	77	21
1	225	62
2	60	17
Child-Pugh score		
5	270	75
6	91	25
Chronic liver disease		
Yes	273	76
No	88	24
Aetiology of chronic liver disease		
HCV	71	20
HBV	33	9
Alcohol	88	24
NASH/NAFLD	72	20
Other	36	10
Unknown	18	5
Cirrhosis		
Yes	240	66
No	120	33
Unknown	1	0.3
Prior treatment		
None	207	57
TACE/TAE	122	34
Surgery	37	10
Ablation,	20	6
Baseline AFP		
<400 ng/mL	222	61
≥400 ng/mL	133	37

*HCV* hepatitis C virus, *HBV* hepatitis B virus, *NASH* non-alcoholic steatohepatitis, *NAFLD* non-alcoholic fatty liver disease, *TAE* transarterial embolisation, *TACE* transarterial chemoembolisation, *AFP* alpha-fetoprotein.

### Radiological and histological evaluation

Imaging characteristics are shown in Table 2. CT was the diagnostic modality in 71%, MRI in 27% and seven patients (2%) had both. The median size of the largest tumour was 6 cm and three patients with prior resection had extrahepatic disease only with no evidence of intrahepatic disease. The majority had three lesions or less, 42% had an extrahepatic disease and 34% had a macrovascular invasion. Of the 361 in the PAC, 120 did not have cirrhosis and 1 was unknown, leaving 240 that were evaluable by NIRC (Fig. 1). The NIRC was fulfilled for 164 of the 240 patients. The remaining 76 patients did not meet the criteria; 43 did not demonstrate arterialisation, 64 did not demonstrate portal venous washout and in six cases the imaging was not evaluable due to missing sequences. As shown in Table 3, prior histological diagnosis of HCC was available for 45 patients and 270 underwent a new tumour biopsy, of which 237 confirmed HCC, and thus giving a total of 282 patients with HCC. Although HCC accounted for 93% of the histological diagnoses, a variety of other diagnoses were made, including cholangiocarcinoma (CC), combined HCC (cHCC)-CC, benign lesions and ‘others’, which included neuroendocrine and breast carcinoma (Fig. 1 and Table 3). Biopsy was non-diagnostic in 13 patients and was not performed in an additional 45 patients due to poor accessibility, low platelets, clotting derangement or ascites (Table 3). In 25% the reason for not performing a biopsy was not given. The NIRC are only applicable to those with cirrhosis, and in order to calculate the specificity and positive predictive value (PPV) of the NIRC using histology as the gold standard, those with cirrhosis and those meeting NIRC were separated as shown in Table 4. CT and MRI were analysed both together and separately. Overall, the sensitivity for NIRC to correctly identify HCC was 65.4% and was slightly higher for MRI compared to CT at 68.9% and 64.6%, respectively. The overall PPV was 91.4% and again was slightly better for MRI at 93.9% compared with 90.3% for CT. The inclusion of 45 patients with prior histology would tend to overestimate the sensitivity and PPV, so the data were also analysed for those who had fresh biopsy only. Only 11 of 45 with prior histology had imaging that met NIRC, and when these patients were excluded, the overall sensitivity reduced to 63.1% and the PPV was 90.6%. The 120 patients who were not cirrhotic, by definition, did not meet the NIRC and so would be recommended to have a biopsy as per international guidelines. Of these, 55 had lesions demonstrating arterialisation and washout. Overall, 102 (85%) had histological confirmation of HCC, and of the 55 with characteristic enhancement patterns, 46 (82%) had histological confirmation of HCC, 3 had CC, 1 was benign, 1 was non-diagnostic and 4 could not have a biopsy for technical reasons.

**Table 2:** Radiological findings.

Radiological findings	*n* = 361	%
Median size of the largest liver lesion (range) (cm)	6 (0–19)	
No. of lesions		
0–3	185	51.2
>3	164	45.4
NK	12	3.3
Extrahepatic disease	151	41.8
Macrovascular Invasion	121	33.5
Tumour arterialisation	269	74.5
Portal venous washout	230	63.7
Radiological evidence of chronic liver disease	230	63.7
Met NIRC for HCC	164	45.4

*NK* not known.

**Table 3:** Results of biopsy.

	*n* = 361	%
Biopsy		
Yes	270	
No	91	
Reason for not performing biopsy (*n* = 91)		
Prior histology of HCC	45	49.5
Not accessible	9	9.9
Low platelets	6	6.6
Clotting	4	4.4
Ascites	1	1.1
Other	26	28.6
Histology (*n* = 302)		
HCC	282	93.4
HCC (new biopsy)	237	78.5
HCC (prior histology)	45	14.9
Cholangiocarcinoma (CC)	11	3.6
Combined HCC-CC	2	0.7
Benign	1	0.3
Other	6	2.0
Non-diagnostic	13

**Table 4:** Concordance of radiology and histology.

	Total			Cirrhotic			NIRC met		
	All^a^	CT	MRI	All^b^	CT	MRI	All^c^	CT	MRI
	361	257	97	240	174	62	164	121	41
Histology	303	217	78	191	141	47	128	93	33
HCC	282	201	74	179	130	45	117	84	31
CC	11	8	3	7	6	1	6	5	1
HCC-CC	2	1	1	2	1	1	2	1	1
Benign	1	1	0	0	0	0	0	0	0
Other	6	6	0	3	3	0	3	3	0
Total non-HCC	20	16	4	12	10	2	11	9	2
Non-diagnostic	13	9	4	12	9	3	6	6	0
No histology	46	30	13	37	25	12	30	22	8

^a^Seven had CT and MRI.

^b^Four had CT and MRI.

^c^Two had CT and MRI.

#### Adverse events

Among the PAC comprising 361 patients, in which 270 new biopsies were performed, there were two adverse events. In both cases, mild bleeding was reported, but there was no haemodynamic compromise or fall in haemoglobin, and no intervention was required. Among the entire submitted cohort of 418, which included those not eligible for systemic therapy due to Child-Pugh score or PS, there were two additional reports of bleeding among 317 biopsy procedures. In one case, the patient developed a high white count and CRP post biopsy and was found to have 100 mL free fluid on CT, which was compatible with post-biopsy bleeding. No intervention was required, and the patient recovered spontaneously. In the second case, the patient died from post-biopsy haemorrhage. This patient had Child-Pugh score of 7 and an international normalised ratio of 1.34. In summary, for those eligible for sorafenib, the rate of mild bleeding was 0.7% and there were no serious sequelae, but beyond these criteria, the risk of serious complications increases. There were no reports of tumour seeding.

#### Treatment

For the 282 patients with histologically confirmed HCC, 88% went on to receive systemic therapy; 223 (79%) received sorafenib, 26 (9%) received a clinical trial or alternative systemic therapy, 8 (3%) had best supportive care and 7 (2%) declined therapy. For 18 (6%), the patient had transferred to another hospital or treatment was not documented.

## Discussion

To our knowledge, this is the largest analysis of NIRC applied to advanced HCC, in which histology has been used as the gold standard. The patient population evaluated here is typical of that offered systemic therapy in routine practice and clinical trials. Given the criteria for sorafenib eligibility defined by NICE, we confined our central analysis to those with Child-Pugh class A and PS 0–2 (the PAC). Indeed, there is good evidence to support restricting treatment to this subgroup with multiple studies demonstrating the very limited benefit of sorafenib to those outside these criteria [7–9]. Within the PAC population, 76% had at least one cause of chronic liver disease, but only 66% were cirrhotic, and only 45% met NIRC. Hence, 65% would have required confirmatory biopsy to make a confident diagnosis. The overall sensitivity and PPV of NIRC in our population were 65.4% and 91.4%, respectively, and this is comparable with previous studies on early disease. A meta-analysis, including 242 studies in which the radiological criteria were not uniformly defined, reported a per-lesion sensitivity and PPV of 73.6% and 85.8%, respectively, for CT and 77.5% and 83.6%, respectively, for gadolinium-enhanced MRI [10]. Both sensitivity and PPV were lower in ‘explant-only’ studies where pathological confirmation was available. A more recent meta-analysis evaluating LI-RADS reported a sensitivity of 66% for LI-RADS ≥5 [5]. In early disease, the criteria are generally used to distinguish between benign and malignant disease, whereas in advanced disease, the criteria are being used to differentiate HCC from another malignancy. Whilst PPV reported in our study and others may be acceptable for early-stage disease, it is far from satisfactory in the setting of advanced disease where reliance on NIRC alone will result in around 10% receiving an incorrect diagnosis. In our study, alternative diagnoses including CC, cHCC-CC and a variety of other tumours were confirmed by biopsy, implying that reliance on imaging alone would have resulted in these patients receiving potentially inappropriate therapy. CC also occurs in the context of chronic liver disease and previous studies have reported similar enhancement patterns in both intrahepatic CC and HCC in cirrhotic patients [11]. Our study serves to confirm these findings. Similarly, the overlap between imaging characteristics of cHCC-CC and HCC has recently been highlighted and biopsy has been recommended to improve diagnostic accuracy [12–14].

The most recent version of the EASL guidelines defines the criteria for radiological diagnosis of HCC, but states that ‘upfront liver biopsy and blood sampling is recommended for clinical and diagnostic trials’ [2]. However, histological diagnosis has only been mandatory for a relatively small number of phase III trials [6, 15–18] and the most recently approved agents have been evaluated in clinical trials for which pathological confirmation was not required [19–22]. Given our findings, it is of particular concern that registrational trials, which have not mandated biopsy, may have recruited up to 10% of patients with a diagnosis other than HCC.

An additional factor limiting the adoption of routine biopsy in HCC is the risk of complications, which may include pain, bleeding, tumour seeding and death. Mild bleeding is reported in around 3–4% cases of liver biopsy and severe bleeding requiring transfusion in 0.5% [23]. In a more recent study that included >1100 biopsies, there were four bleeding events accounting for an incidence <0.4% [18]. Mortality is generally considered to be around 1 in 10,000 [23]. The risk of biopsy is considered to be higher in those with underlying cirrhosis, but in our series, the rate of mild bleeding was only 0.7%. One patient with Child-Pugh class B had severe bleeding from which he died, underlining the increased risk in patients with liver dysfunction. Patients with Child-Pugh class B disease were not included in the seminal SHARP trial and are not eligible for systemic therapy according to NICE guidelines. In the field of practice studies, the outcome for patients with Child-Pugh class B disease treated with sorafenib was poor with a median survival of 5.2 months [8], and recent data for nivolumab in this patient population confirms the poor outlook [24]. Needle track seeding was originally reported at 2.7 or 0.9% per year [25], but subsequent large studies have reported lower rates ranging from 0.14 to 0.76% [26–28]. In the context of advanced disease, the risk of seeding is of less concern and in practice was not reported at all in our series.

The lack of tissue from advanced HCC treated in clinical trials and routine practice has limited the capacity for the widespread genomic analysis and biomarker development that has characterised progress in other cancers. Consequently, the majority of publications exploring the genomics of HCC have been conducted on early-stage, resected tumours, many of which occur in non-cirrhotic livers and therefore may not be representative of tumour biology in advanced disease [29–33]. Furthermore, the molecular divergence has been demonstrated in metastatic tumours [34] and the extent of molecular evolution from early- to late-stage disease has not been well defined. The evidence presented here provides a clear rationale to undertake biopsy in patients with suspected advanced HCC and this will help to accelerate our understanding of cancer biology in advanced HCC.

Despite being a large multicentre prospective study, we acknowledge some limitations. First, although all cases were reviewed by a local specialist hepatobiliary MDT, there was no central review of radiology or histology. It is therefore not possible to differentiate between a true failure of the NIRC to correctly diagnose HCC or a local misinterpretation of the imaging. However, the 11 false-positive cases were evenly distributed across four high-volume centres, suggesting that the findings reflect real-life clinical practice rather than attributable to systematic issues with one centre. Second, it is likely that the false-negative rate may have been underestimated if these patients did not undergo biopsy, but this would imply that the true sensitivity is even lower than we report. Finally, only a minority of patients had both CT and MRI, which could have reduced the sensitivity in our study. However, the sensitivity that we report is in line with other studies and the lack of dual imaging does not undermine the key finding that up to 10% of those who met the NIRC did not have HCC.

## Conclusion

We demonstrate that the majority of patients with advanced HCC do not present with diagnostic imaging, and in those who do, up to one in ten will have a diagnosis other than HCC. In patients with a preserved liver function who are eligible for systemic therapy, tumour biopsy proved to be safe and provided a histological diagnosis of HCC or other cancer in 96% of cases. In the era of precision medicine, we recommend that biopsy should be routinely performed in patients with suspected advanced HCC who are potentially eligible for systemic therapy. The adoption of EASL’s recommendations to incorporate tissue sampling into clinical trials is also strongly endorsed.

## Supplementary information

checklist
